# Lionfish (*Pterois miles*) blow directed jets of water to manipulate prey behavior

**DOI:** 10.1093/beheco/arag087

**Published:** 2026-07-24

**Authors:** Davide Bottacini, Marije Ridder, Emil Bathow, Grigorios Skouradakis, Thanos Dailianis, Maria Mastoraki, Stavros Chatzifotis, Alexander Kotrschal

**Affiliations:** Behavioural Ecology Group, Wageningen University, Wageningen, De Elst 1, 6708 WD, The Netherlands; Behavioural Ecology Group, Wageningen University, Wageningen, De Elst 1, 6708 WD, The Netherlands; Behavioural Ecology Group, Wageningen University, Wageningen, De Elst 1, 6708 WD, The Netherlands; Institute of Marine Biology, Biotechnology and Aquaculture, Hellenic Centre for Marine Research, Gournes Pediados, Heraklion 71500, Greece; Institute of Marine Biology, Biotechnology and Aquaculture, Hellenic Centre for Marine Research, Gournes Pediados, Heraklion 71500, Greece; Institute of Marine Biology, Biotechnology and Aquaculture, Hellenic Centre for Marine Research, Gournes Pediados, Heraklion 71500, Greece; Institute of Marine Biology, Biotechnology and Aquaculture, Hellenic Centre for Marine Research, Gournes Pediados, Heraklion 71500, Greece; Behavioural Ecology Group, Wageningen University, Wageningen, De Elst 1, 6708 WD, The Netherlands

**Keywords:** predation ecology, invasion ecology, prey manipulation, rheotaxis, jet-blowing

## Abstract

Predation is pervasive in the natural world and shapes evolution of predators and prey. Numerous behavioral adaptations allow predators to manipulate prey behavior, resulting in higher success in predator–prey interactions. The lionfishes *Pterois miles* and *Pterois volitans* are highly effective predators and their invasiveness is attributed to their high hunting success. How lionfish attain such a high predation effectiveness remains enigmatic. Lionfish are stalking predators and show a peculiar behavior while approaching their prey fishes: they actively blow directed jets of water at them. This is the only known example of a predator blowing jets of water at mobile prey underwater. The causes and consequences of jet-blowing remain unknown. Here, we tested the hypothesis that lionfish use jets of water to re-orientate their prey and facilitate head-first capture. We conducted 2 separate experiments with 2 different groups of lionfish and prey species. We show that lionfish catch more prey head-first when blowing and that prey exposed to jets orientate towards an approaching lionfish, with smaller lionfish being more likely to blow. However, blowing was not determined by the initial orientation of prey and was largely driven by lionfish size. In addition, in one of the experiments, blowing lionfish struck from a further distance, possibly due to prey swimming towards them in response to blowing. Our results show that jet-blowing allows lionfish to manipulate the behavior of their prey by re-orientating them to facilitate swallowing, providing the first evidence that jet-blowing can be used to manipulate the behavior of mobile prey.

## Teaser

Lionfish are predators that show a very interesting behavior while they approach their prey: they blow water at them. This seems counterintuitive, since the current could easily alert the prey of the presence of the predator. In our experiment, we studied why lionfish do this for the first time. We show that they blow water to orientate their prey and swallow them more easily.

## Introduction

Predation is wide-spread in the natural world and has a major role in shaping animal evolution and ecosystem functioning (Reznick and Endler 1982; Magurran 1990; Hammerschlag et al. 2019). Predators and prey are involved in evolutionary arms races and constantly adapt to increase success in their interactions (Dawkins and Krebs 1979; Brönmark and Miner 1992; Price et al. 2015). There are prominent examples of behavioral adaptations that allow predators to increase their success by manipulating their prey before capture. For instance, archerfish (*Toxotes jaculatrix*) blow jets of water through the air to make their insect prey fall from branches above the water level or underwater to expose hidden food items, triggerfishes (Balistidae) turn sea urchins upside down to expose their more vulnerable body parts and pufferfishes (Tetraodontidae) blow water to excavate prey buried in the sand (Frazer et al. 1991; Wainwright and Turingan 1997; Gerullis and Schuster 2014; Dewenter et al. 2017). In these examples, manipulation physically displaces prey to facilitate capture or consumption. However, predatory fishes can also manipulate their preys' behavior. For instance, anglerfishes (Lophoioidei) lure prey by moving a bioluminescent dorsal fin ray (Maile 2023) and the Livingston's cichlid (*Nimbochromis livingstonii*) mimics a dead fish at the bottom of lake Malawi to attract cichlid prey (McKaye 1981). In these latter examples, a prey is lured within striking distance by a predator body part or the whole animal mimicking a food source.

Fish predators tend to swallow prey head-first to reduce swallowing time and the risk of prey escape (Morris et al. 1956; Reimchen and Douglas 1984; Hoyle and Keast 1987; Reimchen 1991). The fin rays of many bony fishes have evolved into defensive spines that point towards their tail (Price et al. 2015). If swallowed head-first, these spines bend backwards along the body of the prey while, if swallowed tail-first, defensive spines are more likely to bend forward and get stuck in the predator's mouth, inflicting injuries and increasing swallowing time (Reimchen 1991). Head-first swallowing is wide-spread among piscivores and in terrestrial predators it often involves post-capture prey manipulations to orientate a prey fish head-first before swallowing (Reimchen and Douglas 1984; Reimchen 1991). However, most predatory fishes are limited in post-capture manipulations. This is because they can only use their mouth to manipulate their prey and they can often not re-orientate their prey without releasing it, which increases the risk of escape (Morris et al. 1956; Hoyle and Keast 1987). Therefore, predatory fishes tend to catch their prey fishes in a head-first position, a manner that requires no post-catch handling to swallow them head-first (Deelder 1951; Morris et al. 1956). Swallowing prey head-first tends to be more important when the size is of a prey is large relative to that of the predator (Reimchen 1991; Price et al. 2015).

The lionfishes *Pterois miles* and *Pterois volitans* (hereafter, lionfish) have a unique hunting strategy: they slowly stalk their prey with their pectoral fins fully flared for a long time before striking and catching them through water suction created by opening and protrusion of their mouth (Muller et al. 1982; Green et al. 2011; Cure et al. 2012; Côté and Smith 2018; D’Agostino et al. 2020). Lionfish are highly invasive and prey naïveté has a role in their success as predators in their invasive ranges (Cure et al. 2012; Côté and Smith 2018; D’Agostino et al. 2020; Bottacini et al. 2024). However, as lionfish show the same hunting strategy in their native range, prey naïveté alone cannot explain the evolution of their stalking hunting strategy (Cure et al. 2012; McCormick and Allan 2016; Côté and Smith 2018). Lionfish are large and conspicuous and it is unlikely that they can become inconspicuous through camouflage (Marshall et al. 2019; Bottacini et al. 2025). It therefore remains unclear how such a conspicuous animal manages to slowly approach its prey without triggering an antipredator response (Côté and Smith 2018).

Lionfish blow directed jets of water at their prey fish while they approach them (Albins and Lyons 2012; Cure et al. 2012). This is, to our knowledge, the only example of a predator blowing water jets at prey that could flee from them (Albins and Lyons 2012). Jet-blowing has only been reported on lionfish approaching fishes (Albins and Lyons 2012), although lionfish are known to also feed on invertebrates, especially when young (Valdez-Moreno et al. 2012; Côté and Smith 2018; Zannaki et al. 2019). It has been suggested that lionfish blow at their prey to orientate them head-first for more efficient swallowing, as fishes tend to orientate themselves towards the source of a current (positive rheotaxis) and could therefore orientate to face the source of the water jet (Arnold 1974; Albins and Lyons 2012). Alternatively, blowing may allow lionfish to stun their prey by overstimulating their lateral line (Albins and Lyons 2012). These 2 hypotheses are not mutually exclusive; blowing may both orientate prey for head-first capture and reduce anti-predator behavioral responses through overstimulation of a prey's lateral line. Studies investigating these hypotheses are lacking and the causes and consequences of jet-blowing in lionfish remain unknown.

Here, we tested the hypothesis that lionfish blow jets of water at their fish prey to re-orientate them and increase the chance of head-first capture. We offered live prey to lionfish in a controlled environment and analyzed their interactions through video analysis. We predicted that (i) when blowing, lionfish would capture a greater proportion of prey head-first compared to when not blowing, (ii) lionfish would blow more frequently at prey that are not orientated towards them and that (iii) smaller lionfish would be more likely to blow because they were dealing with relatively larger prey. In addition, we predicted that (iv) prey that are blown at would turn to orientate themselves towards an approaching lionfish. On the contrary, prey that is not blow at should not change their orientation during an approach. Our predictions are based on the available knowledge on prey handling in piscivorous predators and rheotaxis in fishes. Our hypotheses assume that swallowing prey head-first allows lionfish to prevent their defensive spines to interfere with swallowing, which should be more problematic for relatively small predators.

## Materials and methods

We conducted 2 separate experiments, using 2 different species of prey: European seabass (*Dicentrarchus labrax*, hereafter, seabass) and gilt-head seabream (*Sparus aurata*, hereafter, seabream). We conducted the first experiment in July 2024 (seabass experiment) and the second one in October–November 2024 (seabream experiment). The experiments were done with 2 distinct groups of lionfish. We used the same experimental apparatus and procedures and treated the animals in the same manner in the 2 experiments, unless stated otherwise.

### Animals and husbandry

**Figure 1:**
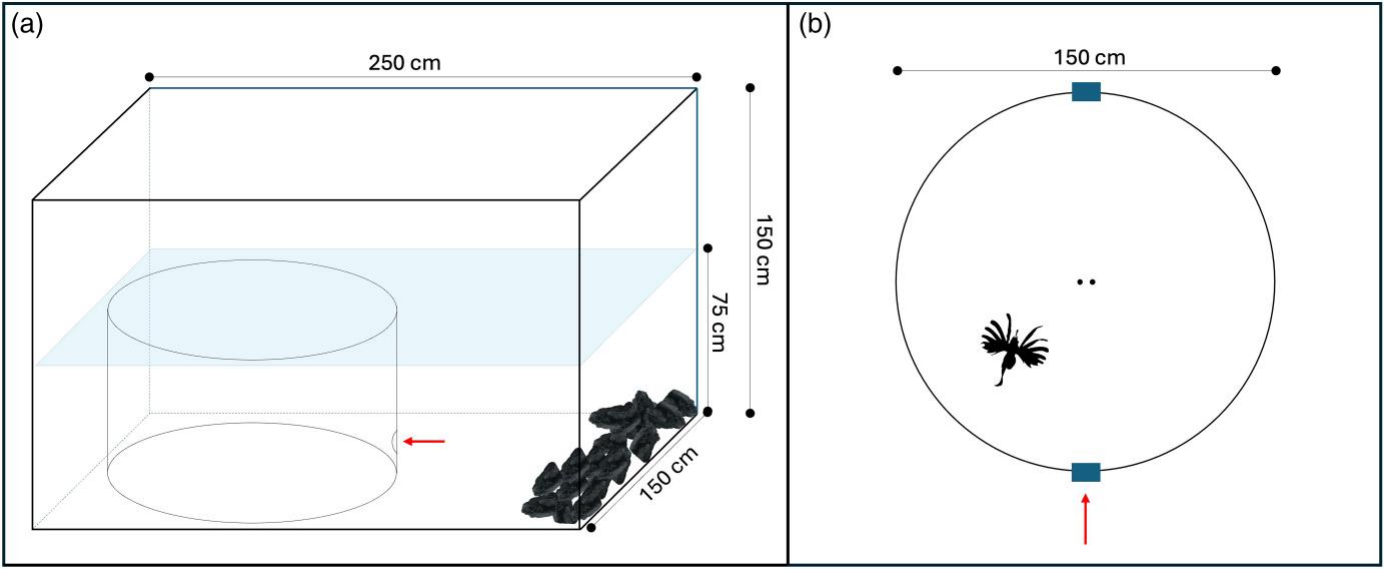
Overview of the set-up for behavioral observations on lionfish–prey interactions. Panel a) shows the communal tank and panel b) shows the top-view of the experimental arena. Arrows show the entrance to the arena. In panel a), the horizontal surface indicates the water level during the trials and the rocks show the location of the enrichment. In panel b), the 2 dots at the centre of the arena represent the screws that we used for scale and the 2 rectangles show the position of the 2 GoPro cameras.

We caught lionfish (*P. miles*) from the coastal waters of Crete (Greece), where they are well-established as an invasive species (Bottacini et al. 2024). We did this using a combination of SCUBA and free diving with 2 hand nets to a maximum depth of 18 m, without anesthetizing the fish. This resulted in 2 lionfish groups with a wide range of sizes (27 lionfish in the seabass experiment, from 11.4 to 32.3 cm in total length and 20 lionfish in the seabream experiment, from 15.9 to 24.4 cm in total length). Lionfish were stocked in a rectangular communal tank (2.5 m × 1.5 m × 1.5 m) at the Hellenic Center for Marine Research, Gournes, Greece. The tanks had a constant inflow of water (temperature: 20 to 22 °C, salinity: 35 mg/L, pH: 7.4) provided by a borehole and a constant outflow which maintained the water level constant (during stocking, 1.36 m of depth). We placed rocks as enrichment along one of the short sides of the tank and a cylindrical fiberglass arena close to the other short side (Fig. 1a). At capture, we assigned an identity to each lionfish through pictures of their unique barred lateral pattern and measured their total lengths to the closest millimeter (Chaves et al. 2016; Phillips and Kotrschal 2021). The lionfish were fed daily with seabass (5 to 9 cm in total length) in the first experiment and gilt-head seabream (3 to 7 cm in total length) in the second experiment. Seabass and seabream were provided by a local hatchery facility and stocked in a separate cylindrical tank that received water from the same source as the lionfish tanks. Seabass and seabream were fed daily with commercial pellet and were also used as prey during the experiments.

### Experimental set-up and procedures

The trials took place inside the experimental arena (150 cm in diameter and 75 cm in height, Fig. 1b). The arena had a circular hole (entrance) of 10 cm in diameter located at 10 cm above the bottom of the tank. Through the entrance, lionfish could access the arena and participate in a trial. The bottom of the arena was covered with a layer (2 cm) of fine sand, which allowed for a uniform color and high contrast between fish and bottom. We placed 2 bolts at 8 cm from each other at the center of the arena, to have a size reference for subsequent video analyses. Every trial was filmed by 3 cameras: a Sony HDR-CX625 (9.2 megapixels, 16:9) fixed above the lionfish tank (2.5 m from water level) and 2 GoPro Hero 7 Black cameras (filming settings: 2.7 K, 60 FPS, linear) fixed at the top of the arena just below the water surface. The Sony camera could film the entire arena and was used to track the movement and orientation of lionfish and prey during trials. Footage from the 2 GoPro cameras was used to determine whether lionfish were blowing.

During the acclimation phase (at least 2 wk) and when not running trials, lionfish could freely enter the arena from the entrance or from above. At the beginning of an experimental day, we lowered the water level of tank to the height of the arena (75 cm), so that it could not be entered from above. We then closed the entrance through a transparent trap door so that no lionfish could enter and placed a prey at the center of the arena. After 1 min of prey acclimation, we opened the door and waited for a lionfish (focal lionfish) to enter. Lionfish decided whether to participate in a trial and were often observed waiting outside the arena for the door to be opened. As soon as the focal lionfish entered the arena, we closed the entrance with the door. We checked that this operation did not interfere with the focal lionfish or prey behavior in pilot trials. A trial started when the focal lionfish entered the arena and ended when the lionfish captured the prey, lost interest or the prey escaped from a strike. We only analyzed trials in which the prey was not moving when a lionfish started swimming towards it in a hunting posture. This was done to minimize changes in lionfish approach angle caused by moving prey. At the end of a trial, the focal lionfish was filmed on either side of the body with one of the GoPro cameras for later identification and gently guided outside the arena.

### Video analyses

We analyzed videos of the trials to measure the behavior and orientation of lionfish and prey during a lionfish approach. An approach started when the focal lionfish maintained a visual orientation of ≤90° towards the prey for 5 s or longer. With this condition, we ensured that the variables that we scored were part of a predator–prey interaction, with the focal lionfish specifically targeting the prey. This excluded the time during which a lionfish simply explored the arena. An approach ended when the lionfish captured the prey, lost interest or the prey escaped from a strike. Therefore, there was only one approach within each trial. We defined loss of interest when a lionfish was not visually directed towards the prey within an angle of 90° for more than 5 s. We discarded videos in which we detected poor quality of the footage or malfunctions of the apparatus during the analysis (<10% in both experiments). We only analyzed approaches in which the prey did not swim more than its own body length during an approach (88% in the seabass experiment and 60% in the seabream experiment). This resulted in 167 videos in the seabass experiment and 215 videos in the seabream experiment. Videos were analyzed using the software Tracker (version 6.1.6, January 2024).

**Figure 2:**
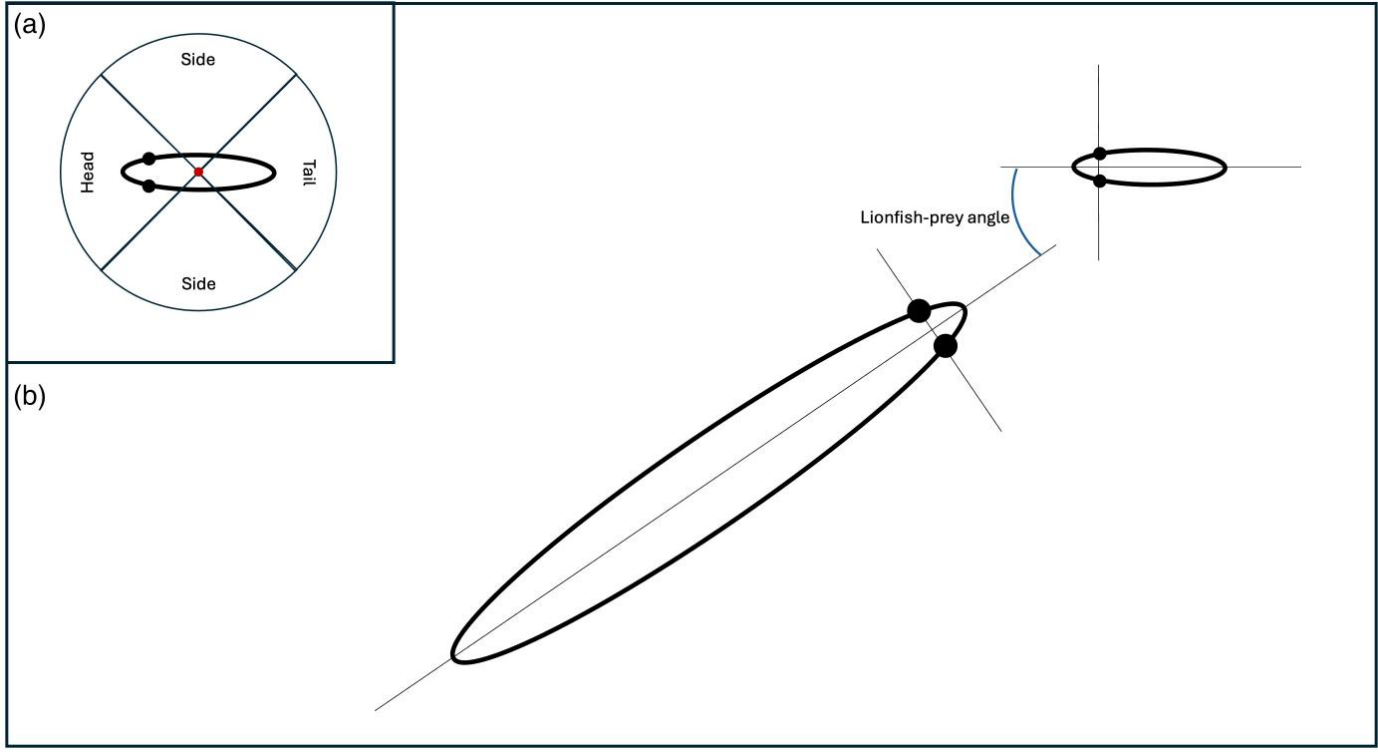
Overview of the variables scored from videos on lionfish and prey orientation. Panel a) shows the definition of catch orientation and panel b) shows the definition of lionfish–prey angle. Oval shapes represent prey (small ovals) and lionfish (large oval), with black dots representing the animals' eyes. In panel a), the dot represents the prey's body center and the 4 quadrants represent the angles that were used to score whether a prey was caught head-, tail, or side-first. In panel b), the thin straight lines mark the longitudinal and transversal body axes of the animals and the curved line marks the lionfish–prey angle. A screenshot from one of the videos used during data collection is available in the Figure S2.

For each approach, we recorded lionfish identity, prey size, blowing behavior, strike success, catch orientation, strike distance, lionfish–prey angle. Lionfish identity was based on the unique lateral pattern of lionfish individuals and was determined by analyzing the lateral videos filmed at the end of every trial (Chaves et al. 2016; Phillips and Kotrschal 2021). Prey size was measured with a ruler to the closest millimeter before placing the prey in the arena. Blowing behavior (present or absent) was defined based on the characteristic rhythmic moving pattern of the gill covers during an approach (Albins and Lyons 2012). Strike success was determined based on whether a lionfish caught the prey (success) or not (failure). Catch orientation was defined as head-first, side-first or tail-first depending on the orientation at which the prey was caught (Fig. 2a). Strike distance was defined as the distance between the tip of the lionfish snout and the closest part of the prey at the moment of strike. Lionfish–prey angle was defined as the absolute angle between 2 lines perpendicular to the lines connecting the eyes of the animals (Fig. 2b). Lionfish–prey angle was measured at start of an approach (start angle) and at the moment a lionfish struck (end angle). Data from the seabream experiment showed that most of the change in angle during an approach is due to the prey changing orientation, while lionfish tend to approach following a straight trajectory (Figure S1). The outcome of a strike was recorded as a success or failure based on whether the prey was caught or not.

### Statistical analyses

Data from the 2 experiments were always modeled separately. We used R 4.4.1 (R Core Team 2024) for all statistical analyses and data visualizations. We fitted generalized linear mixed models (GLMMs) using the package “glmmTMB” (Brooks et al. 2017), choosing distributions and link functions based on the nature of our response variables and adjusting them when necessary to meet model assumptions (Santon et al. 2023). We accounted for multiple observations on the same individual lionfish by adding lionfish identity (Lionfish_ID) as a random factor in every model. We included interaction terms when they were biologically meaningful (Santon et al. 2023). We did not account for date or order of trials for each individual, since there was no obvious pattern in the participation rate of lionfish throughout the studies (Figures S3 and S4). We checked for violations of the model assumptions using the package “DHARMa” (Hartig 2022). We extracted test statistics and *P* values with the package “car” (Fox et al. 2001) and we interpreted differences as statistically significant when their associated *P* value was lower than 0.05 or when their 95% confidence interval did not overlap with 0. Full summary tables for each model are available in the Tables S1–S5. We used the package “tidyverse” (Wickham et al. 2019) for data visualizations.

To test whether blowing increased the probability of head-first captures, we tested whether the proportion of head-first captures differs between approaches with blowing and without blowing (Catch_orientation ∼ Blowing + (1|Lionfish_ID), family = binomial). We defined nonhead-first catches as the sum of side- and tail-catches. To investigate what factors predict whether a lionfish blew during an approach, we modeled the proportion of blowing approaches as a function of absolute lionfish–prey start angle and lionfish size (Blowing ∼ Start_angle + Lionfish_size + (1|Lionfish_ID), family = binomial). To test the effect of blowing on prey orientation, we modeled the change in lionfish–prey angle during an approach as a function of blowing and start angle (Angle_change ∼ Blowing + Start_angle + Blowing:Start_angle + (1|Lionfish_ID), family = gaussian). We defined a change in lionfish–prey angle as the difference between the absolute start angle and the absolute end angle. Therefore, positive values of change in angle indicate a net change of the prey orientation towards a lionfish, while negative values indicate a net change in orientation away from a lionfish. Based on our prediction, we expected no change in angle in approaches without blowing and a change in angle equal to the initial angle in approaches with blowing. We modeled strike distance as a function of blowing to test for the possibility that lionfish could strike from a further distance in approaches with blowing (Strike_distance ∼ Blowing + (1|Lionfish_ID), family = gamma in the seabass experiment and family = gaussian in the seabream experiment). To test whether blowing increases lionfish hunting success, we modeled the strike success rate as a function of blowing (Strike_outcome ∼ Blowing + (1|Lionfish_ID), family = binomial). Because there were no failures in the seabream experiment, we only modeled strike success in the seabass experiment.

### Ethical note

The experimental protocol for the maintenance of wild lionfish in captivity and experiments on their capture of live prey was approved by the ethics committee of the Hellenic Center for Marine Research (decision number: 125/12.09.2023).

## Results

**Figure 3:**
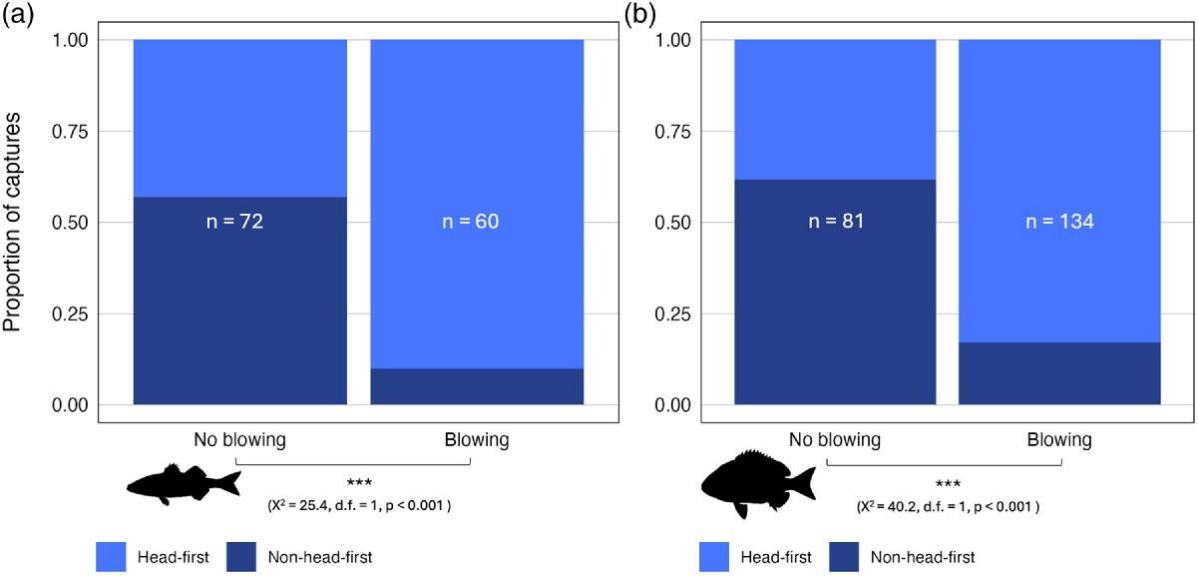
Effect of blowing on the proportion of head-first captures. Proportion of head-first captures in the seabass (panel a) and seabream (panel b) experiment in blowing and nonblowing trials (*n* indicates the sample size). Nonhead-first captures include both lateral captures and tail captures.

**Figure 4:**
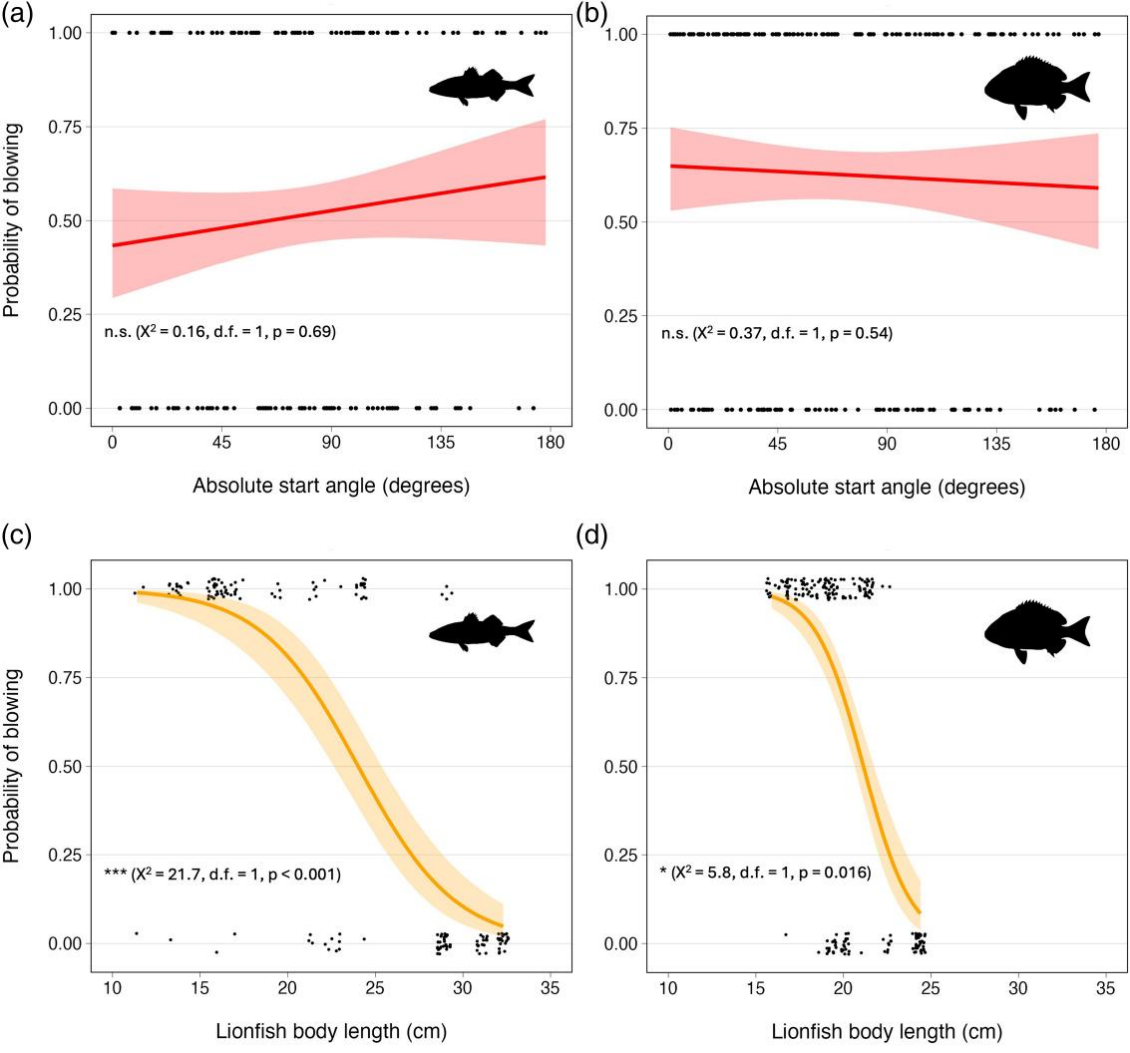
Effect of lionfish–prey start angle and lionfish length on blowing probability. Probability of a lionfish blowing as a function of absolute lionfish–prey (panels a, b) start angle and lionfish total length (panels c, d) in the seabass and seabream experiment. Absolute start angle varies from 0° (prey facing a lionfish) and 180° (prey orientated in the complete opposite direction as a lionfish). Dots represent (jittered) raw data points.

**Figure 5:**
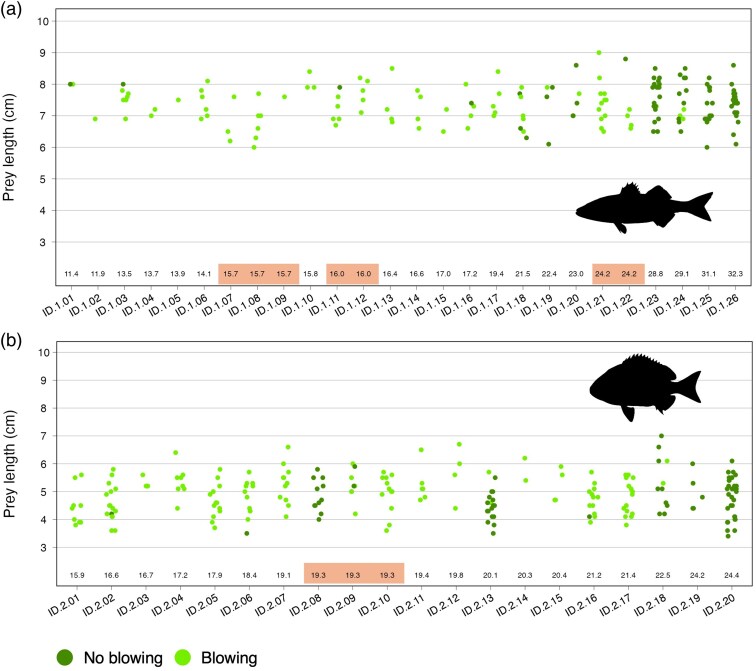
Individual variation in blowing behavior among lionfish. Differences in blowing behavior among lionfish individuals when presented with similar-sized prey in the seabass (panel a) and seabream (panel b) experiment. Dots represent (horizontally jittered) raw data points of prey sizes offered to each lionfish, color-coded based on whether a lionfish blew at them. The horizontal axes show the identities of the lionfish participating in trials. The numbers along the horizontal axes associated with each identity show the total length of the lionfish in cm. Lionfish of the same total length within each experiment are highlighted.

**Figure 6:**
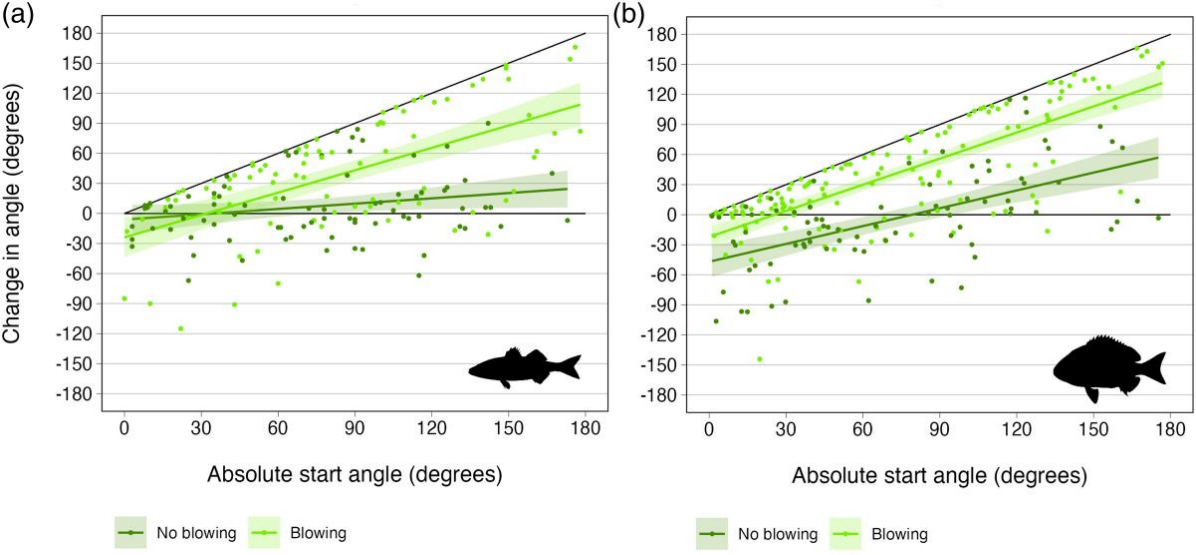
Effect of blowing on lionfish–prey angle. Absolute change in lionfish–prey angle during approaches in the seabass (panel a) and seabream (panel b) experiment. Horizontal axes show the absolute lionfish–prey start angle (0° represents prey and lionfish facing each other and 180° represents a prey orientated in the complete opposite direction as a lionfish). Positive changes in lionfish–prey angle represent changes in prey orientation towards a lionfish, while negative changes represent changes in orientation away from a lionfish. Black lines show the predicted response when prey were blown at (β_1_ = 1) and the predicted response when prey were not blown at (β_1_ = 0). Dots represent raw data points and shaded areas around the lines represent 95% confidence intervals.

**Figure 7:**
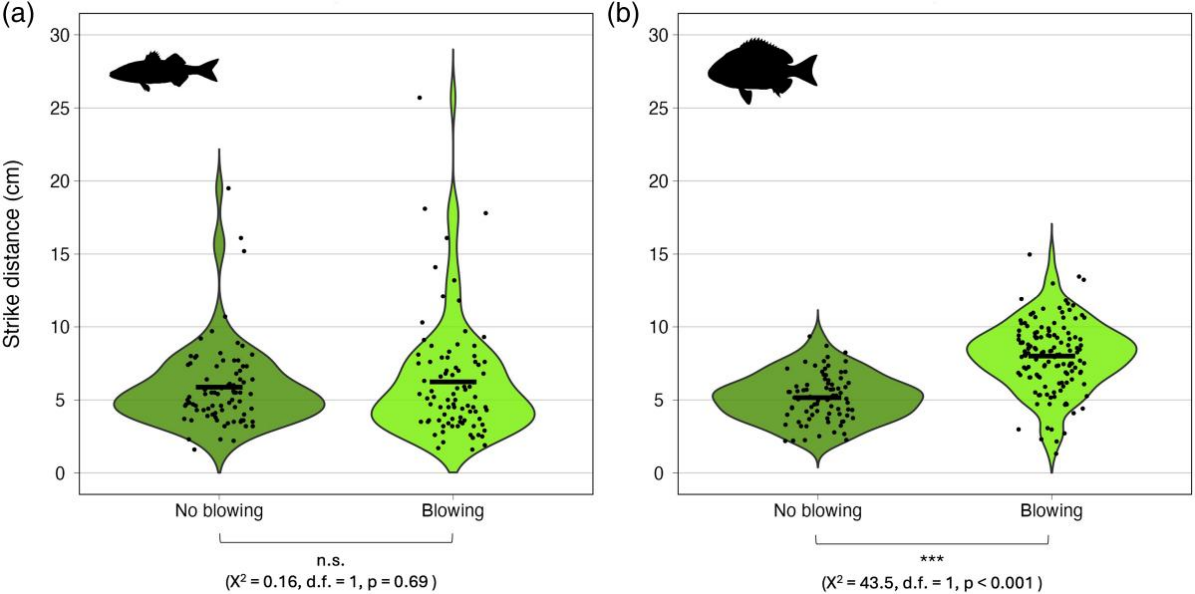
Effect of blowing on strike distance. Strike distance in the seabass (panel a) and seabream (panel b) experiment in blowing and nonblowing trials. Strike distance was defined as the distance between lionfish and prey at the moment a lionfish struck. Horizontal bars in the violin plots represent means and dots represent (horizontally jittered) raw data points.

**Figure 8:**
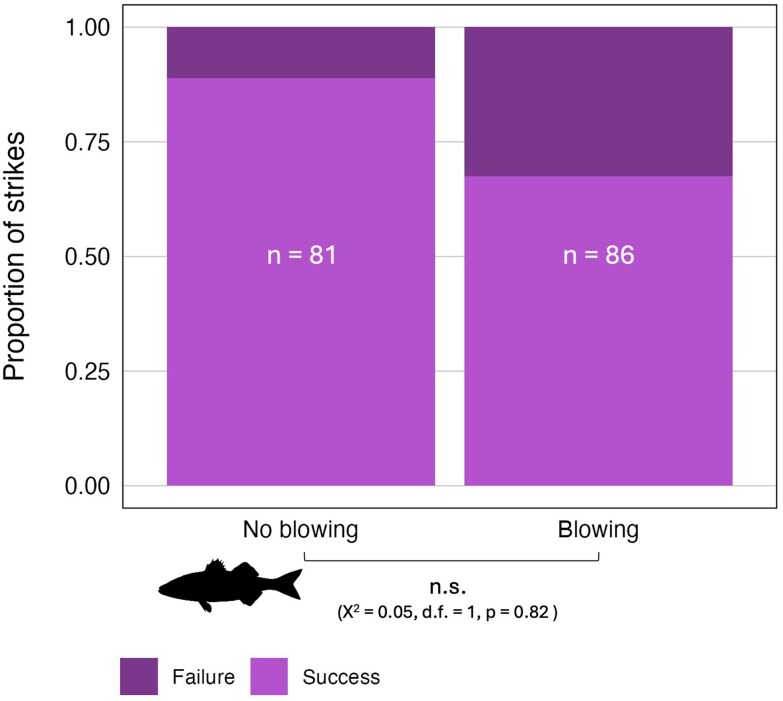
Effect of blowing on lionfish strike success rate. Proportion of successful and unsuccessful strikes in blowing and nonblowing approaches in the seabass experiment (*n* indicates the sample size). There were no failures in the seabream experiment.

Jet-blowing had a strong effect on the proportion of head-first captures in both experiments (Fig. 3, GLMM, χ^2^ = 25.4, df = 1, *P* < 0.001 in the seabass experiment and χ^2^ = 40.2, df = 1, *P* < 0.001 in the seabream experiment). When a lionfish blew, a prey was 11.9 (95% CI: 4.5, 31.2) and 7.8 (95% CI: 4.14, 14.7) times more likely to be caught head-first than when the lionfish was not blowing in the seabass and seabream experiments respectively. The angle between lionfish and prey at the start of an approach had no effect on whether a lionfish blew in either the seabass or seabream experiment (Fig. 4, GLMM, χ^2^ = 0.16, df = 1, *P* = 0.69 in the seabass experiment and χ^2^ = 0.37, df = 1, *P* = 0.54 in the seabream experiment). Large lionfish were less likely to blow than small ones in both the seabass (Fig. 4, GLMM, χ^2^ = 21.7, df = 1, *P* < 0.001) and seabream (Fig. 4, GLMM, χ^2^ = 5.8, df = 1, *P* = 0.016) experiment. The majority of the lionfish that participated in our experiments blew at least once: 23 of 26 in the seabream experiment and 17 out of 20 in the seabass experiment (Fig. 5). In the seabass experiment, prey turned towards a lionfish when they were approached with blowing. The angle between lionfish and prey did not change as a function of the initial angle in approaches without blowing (Fig. 6, β_1_: 0.18, 95% CI: −0.02, 0.38) while it did when lionfish blew (Fig. 6, β_1_ = 0.74, 95% CI: 0.56, 0.92). In the seabream experiment, prey turned towards a lionfish in approaches with or without blowing, but the change in orientation was larger when a lionfish blew (Fig. 6, β_1_ = 0.87, 95% CI: 0.74, 0.99 in blowing approaches and β_1_ = 0.59, 95% CI: 0.42, 0.76 in approaches without blowing). Strike distance was not affected by blowing in the seabass experiment (Fig. 7, GLMM, χ^2^ = 0.16, df = 1, *P* = 0.69), while it was longer when lionfish blew in the seabream experiment (Fig.7, GLMM, χ^2^ = 43.5, df = 1, *P* < 0.001, effect size: 3.1 cm, 95% CI: 2.20 cm, 4.04 cm). In the seabass experiment, the strike success rate was not different between blowing and nonblowing approaches (Fig. 8, GLMM, χ^2^ = 0.04, df = 1, *P* = 0.82). In the seabream experiment, the strike success rate was 100% in both blowing and nonblowing approaches.

## Discussion

We tested possible causes and consequences of jet-blowing in lionfish. We predicted that blowing would result in a higher proportion of head-first capture by decreasing lionfish–prey angle through prey re-orientation. The proportion of head-first captures was higher in trials where lionfish blew at their prey, supporting this prediction. The strike success rate was not different between blowing and nonblowing approaches, suggesting that blowing does not increase the hunting success of lionfish, but probably provides post-capture benefits such as easier swallowing. Prey that was blown at turned towards the approaching lionfish, showing that blowing is used to reduce the lionfish–prey angle. Similar responses can be expected in a variety of lionfish prey, since positive rheotaxis is highly conserved across taxa in fishes (Arnold 1974; Coombs et al. 2020). Lionfish produce jets of water in pulses (Albins and Lyons 2012), which resulted in the same behavioral response in prey (ie, positive rheotaxis) that would be expected if fish were reacting to a current with constant speed, which is more common in natural and experimental contexts (Arnold 1974). In the seabass experiment, there was no change in orientation when lionfish approached prey without blowing. However, in the seabream experiment prey turned towards a lionfish when they were approaching without blowing, although the change in angle was lower than in approaches with blowing. This could be interpreted as the result of predator inspection or as part of more general exploratory behavior towards a novel object entering a simple, empty arena (Dugatkin 1992). Alternatively, it is possible that the water movement generated by lionfish swimming with their flared pectoral fins is sufficient to generate a response in prey. The different response by prey to nonblowing lionfish in our experiments could be due to species-specific differences or size differences between prey in the 2 experiments. Targeted behavioral assays of seabream and seabass juveniles are needed to explore the potential of such species-specificities underlying the subtle differences we observed.

Our first prediction on the causes of jet-blowing was that lionfish would blow more often with large absolute lionfish–prey start angles. Our results do not support this prediction. This is especially surprising considering the results showing that blowing contributes to prey reorientation. We predicted that blowing would be most advantageous when prey are not orientated towards them because in that case a change in prey orientation should have a larger effect on how easy it becomes to swallow. Conversely, considering that blowing probably has a metabolic cost (Schlegel et al. 2006), lionfish should prefer not to blow if prey do not need to be reoriented. Our second prediction on the causes of jet-blowing was that larger lionfish would be less likely to blow. Our results show that lionfish size had a large influence on whether a lionfish blew during an approach, with small lionfish blowing more frequently than large ones. This confirms previous observations indicating that blowing lionfish are generally smaller than nonblowing ones (Albins and Lyons 2012). Prey size was held constant regardless of lionfish size in our experiments. Therefore, larger lionfish were dealing with relatively smaller prey and facing a lower risk of injuries during swallowing, decreasing potential benefits of blowing (Reimchen 1991; Price et al. 2015). Our data provides tentative support to the hypothesis that the ratio between lionfish and prey size could be more important than absolute lionfish size; in the seabream experiment, we used slightly smaller prey (4.9 cm vs 7.4 cm). This could explain why the largest lionfish blowing in the seabream experiment was smaller (22.5 cm) than the largest lionfish blowing in the seabass experiment (29.1 cm). As the variation in lionfish size was much larger than that in prey size, it was mostly lionfish that drove this ratio in our experiments. It was therefore not possible to test for any effect of lionfish/prey size ratio on blowing.

The effect of lionfish size on blowing could also be interpreted in terms of differences in return of energetic investment between small and large lionfish. This is based on the assumption that there is a metabolic cost associated with blowing (Schlegel et al. 2006). Prey size being equal, a successful strike will give a large lionfish a relatively lower energetic benefit, which may not justify the cost of blowing while approaching. This is especially relevant considering that any unit increase in mouth diameter should result in a cubic increase in the volume of blown water and, therefore, a cubic increase in the amount of energy needed to produce a water jet with the same velocity. To study this tradeoff, we would need to quantify the exact costs of blowing and swimming in hunting lionfish. Regardless of the exact costs and benefits involved, if blowing is determined by the principle of return of investment, the ratio between lionfish and prey size, rather than absolute predator size, could be a better predictor of whether lionfish blow. Future experiments with strategic combinations of lionfish and prey sizes could be conducted to test for the effect of the relative size of prey on blowing. When controlling for the ratio between lionfish and prey size, the value in eating a prey can be controlled to be the same regardless of the size of a lionfish. Such experiments will also control for the confounder of larger lionfish having a lower risk of injury while swallowing; if the size ratio is controlled for across lionfish sizes, there can be the same risks of injury while swallowing a prey in smaller and larger lionfish.

The 2 largest lionfish in both our experiments did not blow in any trial, which could suggest that blowing might be a behavior typical of juveniles that is then gradually abandoned during development. The morphology and body proportions of lionfish change significantly as they grow, including in their head and mouth region (Rojas-Vélez et al. 2024). Therefore, it is possible that large lionfish simply lose the ability to blow due to morphological changes in their mouth. However, this is not likely to explain the whole range of variation in blowing in our experiments for 4 reasons. First, several lionfish blew only in some of their approaches, indicating that there are also other factors triggering blowing apart from the mere ability of lionfish to blow. Second, in the seabass experiment we had 2 lionfish around 29 cm in total length that occasionally blew, while the 2 largest lionfish in the seabream experiment never blew although they were considerably smaller (around 24 cm). Although this inference is based on very few individuals, if blowing depended on morphological traits, we would expect a clearer size boundary between blowing and nonblowing lionfish. Third, past observations report blowing in lionfish of estimated lengths of up to 30 cm (Albins and Lyons 2012). Fourth, because jet-blowing probably evolved from coughing, it should require similar buccal movements and there is no reason to hypothesize that large lionfish could lose the ability to cough (Wainwright and Turingan 1997). Therefore, we argue that blowing is within the behavioral repertoire of lionfish of any size. It is noteworthy that we found major individual differences in blowing behavior. These differences are particularly striking when looking at individuals of the same size. For instance, there were 3 lionfish of exactly 19.3 cm in total length in the seabream experiment. One of these blew in all the trials, one blew in none and one blew only occasionally, despite being offered prey of the same species and of comparable size. The trials took place over multiple days, making any effects related to hunger and motivation unlikely to explain these individual differences.

Most of our results were very similar for 2 independent experiments, which were done with 2 different prey species and 2 distinct groups of lionfish. Observations from video analyses showed that prey that is blown at start swimming against the current produced by a lionfish. Based on this observation, we predicted that swimming prey should start moving towards the lionfish when blowing is interrupted at the moment of strike. This could allow blowing lionfish to strike from a further distance. When we tested for differences in strike distance, it was longer in blowing approaches compared to nonblowing ones in the seabream experiment. The mean strike distance in blowing trials (8.2 cm) was very close to 9.6 cm, which is the distance reached by water jets (Albins and Lyons 2012). We did not find the same effect in the seabass experiment. This could simply be explained by differences in swimming speed or reactiveness between the 2 different prey species. What goes against this reasoning is that positive rheotaxis is highly conserved across fishes and one of its main functions is that fishes can swim against a current and avoid displacement (Arnold 1974; Coombs et al. 2020).

Lionfish feed on a variety of prey and have been observed to blow more or less frequently depending on the species that they target (Albins and Lyons 2012; Green and Côté 2014; Zannaki et al. 2019; D’Agostino et al. 2020). Part of this variation could be explained by the presence (or lack) of defensive structures in prey and their body proportions. Deeper-bodied prey is usually more difficult to swallow and the same is true for prey with defensive structures (Brönmark and Miner 1992; Price et al. 2015). In our experiments, both prey species had defensive spines and their body proportions were similar. Future experiments could test for differences between prey with or without defensive spines to test whether lionfish blow more at defended prey. We fed 30 rockpool shrimp (*Palaemon* sp.) to the same lionfish that we tested in the seabream experiment and we observed no blowing. This could be because *Palaemon* shrimp are benthic organisms and could therefore react to currents differently from fishes (Berglund 1982; Carvalho et al. 2017; Deli et al. 2018). For instance, a current might not displace or reorientate them at all or stimulate them to hold tighter to the substrate, making capture by lionfish more difficult. To our knowledge, there is no information on the behavioral responses of *Palaemon* shrimp to currents or their antipredator responses. The different behavior of lionfish towards shrimp could be studied in future experiments to investigate how consistent it is across species invertebrates with different ecology. These experiments should first investigate how the specific species of invertebrates that are tested react to a current.

Alternative hypotheses to prey reorientation may also explain jet-blowing in lionfish. For example, pulsed water jets might momentarily impair prey fishes by overstimulating their lateral line (Albins and Lyons 2012), and lionfish could potentially blow chemical cues that distract or confuse their prey. These hypotheses are not incompatible with the idea that lionfish use jets to reorient prey, nor should they be viewed as mutually exclusive. Multiple mechanisms may operate simultaneously, producing additive effects on prey behavior. However, that lionfish blow chemical cues at their prey seems unlikely, since there is no indication that they may possess specific glands that could serve this purpose (Côté and Smith 2018; Bottacini et al. 2024). To test this hypothesis the chemical composition of blown water needs to be characterized (Chua et al. 2016). We consider the possibility that jets of water can overwhelm their prey lateral line more plausible (Mogdans 2019). A first approach to testing this hypothesis could be measuring the effects of artificially produced jets of water on various behaviors in fishes. Any stunning effect mediated by the lateral line can be expected to interfere with behaviors and functions that go beyond antipredator responses. Such studies could include a manipulative component, where the sensitivity of the fish lateral line is interfered with pharmacologically (McHenry et al. 2009).

Our results suggest that jet-blowing causes behavioral changes in fish prey, initiating swimming and positive rheotaxis in fish that were not moving. To our knowledge, this is possibly the only example of a predator manipulating prey behavior through jet-blowing. While blowing jets of water is a behavior found in many distantly related fishes, it is usually used to displace, turn or dig out immobile prey (Frazer et al. 1991; Wainwright and Turingan 1997; Dewenter et al. 2017). In all these species, jet-blowing likely evolved from coughing, a behavior that is used by fishes to expel voluminous objects from their mouth (Wainwright and Turingan 1997). In addition, positive rheotaxis is extremely widespread in fishes (Arnold 1974; Coombs et al. 2020). Why is jet-blowing not more common among fish predators, then? The answer could lie in the peculiar stalking hunting strategy of lionfish, which allows them to approach prey up to an extremely short distance before striking (Green et al. 2011; Green and Côté 2014). Blowing creates a water current that only reaches an extremely short range (Albins and Lyons 2012). Most predators would be detected and recognized before their prey can be influenced by their water jets. However, this explanation is unlikely since lionfish water jets reach a maximum distance of approximately 10 cm, which is shorter than the approximately 25 cm of mean closest approach distance that naïve prey tolerate in the invaded Mediterranean (D’Agostino et al. 2020). In addition, lionfish blow more often in their native range, where prey are not naïve and should keep a longer distance from an approaching lionfish compared to the invaded western Atlantic (Albins and Lyons 2012; Cure et al. 2012; D’Agostino et al. 2020).

We are the first to report jet-blowing in lionfish from the Mediterranean, showing that this behavior is also present in *P. miles* and is not exclusive of *P. volitans*, the predominant species of invasive lionfish in the western Atlantic (Hamner et al. 2007). Lionfish caught a larger proportion of prey head-first in approaches with blowing. Prey that was blown at actively turned towards an approaching lionfish and started swimming against the current. This makes lionfish the first example of predators using jet-blowing to manipulate the behavior of mobile prey, rather than to physically move or displace immobile ones. Jet-blowing may also allow lionfish to strike on their prey from a closer distance. We observed large variation between and within individual lionfish in blowing behavior. Lionfish did not blow more often on prey that was not orientated towards them. While lionfish size explained some of the variation in blowing behavior observed in our experiments, the factors determining whether lionfish blow while approaching their prey remain largely unknown. We suggest that the ratio between lionfish and prey size, the presence of defensive structures and the body proportions of prey are studied in future experiments on the causes of jet-blowing in lionfish. Such experiments should consider the variation among lionfish individuals and could also include invertebrate prey.

## Supplementary Material

arag087_Supplementary_Data

## Data Availability

Raw data and R script will be made available with the online version of this article. Analyses reported in this article can be reproduced using the data provided by Bottacini et al. (2026).

## Additional permits

Capture of live lionfish by SCUBA and free divers was allowed by the local authorities through a sample collection permit from the Hellenic Ministry for Environment and Energy (permit number: 6ΡΔΛ4653Π8-I9Ρ).
