# Supplementary material for: Lionfish (Pterois miles) blow directed jets of water to manipulate prey behavior
Source: Behav Ecol. 2026 Jul 24;37(5):arag087. doi: 10.1093/beheco/arag087 (PMC13435627; doi:10.1093/beheco/arag087)
Supplement: Supplementary file 1 [file arag087_supplementary_data.zip › Supplementary_Legends.docx]

**Supplementary material**

**Table S1.** Full tables for the models on the **effect of blowing on the proportion of head-first captures** in the seabass and seabream experiment.

| **Seabass experiment** | | | | |
| --- | --- | --- | --- | --- |
| **Family** | binomial (logit) |  |  |  |
| **Formula** | Catch_orientation ~ Blowing + (1 \| Lionfish_ID) | | | |
| **Random Effects** | | | | |
| Groups | Name | Variance | Std. Dev. |  |
| Lionfish_ID | (Intercept) | 2.42E-08 | 0.0001555 |  |
|  |  |  |  |  |
| **Number of observations** | 132 |  |  |  |
| **Lionfish_ID** | 24 |  |  |  |
| **Fixed Effects** | | | | |
|  | Estimate | Std. Error | z value | p value (>\|z\|) |
| Intercept | -0.2796 | 0.238 | -1.175 | 0.24 |
| Blowing | 2.4768 | 0.4918 | 5.037 | 4.74E-07 |
| **Seabream experiment** | | | | |
| **Family** | binomial (logit) |  |  |  |
| **Formula** | Catch_orientation ~ Blowing + (1 \| Lionfish_ID) | | | |
| **Random Effects** | | | | |
| Groups | Name | Variance | Std. Dev. |  |
| Lionfish_ID | (Intercept) | 0 | 0 |  |
|  |  |  |  |  |
| **Number of observations** | 215 |  |  |  |
| **Lionfish_ID** | 20 |  |  |  |
| **Fixed Effects** | | | | |
|  | Estimate | Std. Error | z value | p value (>\|z\|) |
| Intercept | -0.478 | 0.2286 | -2.091 | 0.0365 |
| Blowing | 2.0521 | 0.3236 | 6.341 | 2.29E-10 |

**Table S2.** Full tables for the models on the **effect of lionfish size and initial angle on the proportion of blowing approaches** in the seabass and seabream experiment.

| **Seabass experiment** | | | | |
| --- | --- | --- | --- | --- |
| **Family** | binomial (logit) |  |  |  |
| **Formula** | Blowing ~ Start_angle + Lionfish_size + (1\|Lionfish_ID) | | | |
| **Random Effects** | | | | |
| Groups | Name | Variance | Std. Dev. |  |
| Lionfish_ID | (Intercept) | 2.28E+00 | 0.00016 |  |
|  |  |  |  |  |
| **Number of observations** | 167 |  |  |  |
| **Lionfish_ID** | 26 |  |  |  |
| **Fixed Effects** | | | | |
|  | Estimate | Std. Error | z value | p value (>\|z\|) |
| Intercept | 9.925212 | 2.042696 | 4.859 | 1.18E-06 |
| Start_angle | 0.001426 | 0.003572 | 0.399 | 0.69 |
| Lionfish_size | -0.420333 | 0.090291 | -4.655 | 3.24E-06 |
| **Seabream experiment** | | | | |
| **Family** | binomial (logit) |  |  |  |
| **Formula** | Blowing ~ Start_angle + Lionfish_size + (1\|Lionfish_ID) | | | |
| **Random Effects** | | | | |
| Groups | Name | Variance | Std. Dev. |  |
| Lionfish_ID | (Intercept) | 17.31 | 4.161 |  |
|  |  |  |  |  |
| **Number of observations** | 214 |  |  |  |
| **Lionfish_ID** | 20 |  |  |  |
| **Fixed Effects** | | | | |
|  | Estimate | Std. Error | z value | p value (>\|z\|) |
| Intercept | 36.909325 | 14.799527 | 2.494 | 0.0126 |
| Start_angle | 0.002671 | 0.004362 | 0.612 | 0.5403 |
| Lionfish_size | -1.709735 | 0.712026 | -2.401 | 1.63E-02 |

**Table S3.** Full tables for the models on the **effect of blowing on the change in angle by prey during an approach** in the seabass and seabream experiment.

| **Seabass experiment** | | | | |
| --- | --- | --- | --- | --- |
| **Family** | gaussian (identity) |  |  |  |
| **Formula** | Angle_change ~ Blowing + Start_angle + Blowing :Start_angle + (1\|Lionfish_ID) | | | |
| **Random Effects** | | | | |
| Groups | Name | Variance | Std. Dev. |  |
| Lionfish_ID | (Intercept) | 4.83E-09 | 6.95E-05 |  |
|  |  |  |  |  |
| **Number of observations** | 167 |  |  |  |
| **Lionfish_ID** | 26 |  |  |  |
| **Fixed Effects** | | | | |
|  | Estimate | Std. Error | z value | p value (>\|z\|) |
| Intercept | -6.1152 | 8.7112 | -0.702 | 4.83E-01 |
| Blowing | -17.7783 | 12.266 | -1.449 | 0.1472 |
| Start_angle | 0.1764 | 0.101 | 1.747 | 0.0807 |
| Blowing :Start_angle | 0.5683 | 0.1357 | 4.189 | 2.80E-05 |
|  | Estimate | SE | Lower.CI | Upper.CI |
| **Blowing** | 0.7446949 | 0.09054965 | 0.56722082 | 0.9221689 |
| **No-blowing** | 0.1764283 | 0.10100493 | -0.02153778 | 0.3743943 |
| **Seabream experiment** | | | | |
| **Family** | gaussian (identity) |  |  |  |
| **Formula** | Angle_change ~ Blowing + Start_angle + Blowing :Start_angle + (1\|Lionfish_ID) | | | |
| **Random Effects** | | | | |
| Groups | Name | Variance | Std. Dev. |  |
| Lionfish_ID | (Intercept) | 1.26E-05 | 0.003546 |  |
|  |  |  |  |  |
| **Number of observations** | 214 |  |  |  |
| **Lionfish_ID** | 20 |  |  |  |
| **Fixed Effects** | | | | |
|  | Estimate | Std. Error | z value | p value (>\|z\|) |
| Intercept | -46.97357 | 7.59863 | -6.182 | 6.34E-10 |
| Blowing | 24.74082 | 9.41056 | 2.629 | 0.00856 |
| Start_angle | 0.593 | 0.08688 | 6.825 | 8.76E-12 |
| Blowing :Start_angle | 0.27561 | 0.10839 | 2.543 | 1.10E-02 |
|  | Estimate | SE | Lower.CI | Upper.CI |
| **Blowing** | 0.8686044 | 0.06481532 | 0.7415688 | 0.9956401 |
| **No-blowing** | 0.5929965 | 0.08688006 | 0.4227147 | 0.7632783 |

**Table S4.** Full tables for the models on the **effect of blowing on strike distance** in the seabass and seabream experiment.

| **Seabass experiment** | | | | |
| --- | --- | --- | --- | --- |
| **Family** | gamma (log) |  |  |  |
| **Formula** | Strike_distance ~ Blowing + (1 \| Lionfish_ID) | | | |
| **Random Effects** | | | | |
| Groups | Name | Variance | Std. Dev. |  |
| Lionfish_ID | (Intercept) | 4.15E-02 | 0.2036 |  |
|  |  |  |  |  |
| **Number of observations** | 167 |  |  |  |
| **Lionfish_ID** | 26 |  |  |  |
| **Fixed Effects** | | | | |
|  | Estimate | Std. Error | z value | p value (>\|z\|) |
| Intercept | 1.73719 | 0.08837 | 19.658 | <2e-16 |
| Blowing | 0.04057 | 0.10061 | 0.403 | 6.87E-01 |
| **Seabream experiment** | | | | |
| **Family** | gaussian (identity) |  |  |  |
| **Formula** | Strike_distance ~ Blowing + (1 \| Lionfish_ID) | | | |
| **Random Effects** | | | | |
| Groups | Name | Variance | Std. Dev. |  |
| Lionfish_ID | (Intercept) | 0.9339 | 0.9664 |  |
|  |  |  |  |  |
| **Number of observations** | 214 |  |  |  |
| **Lionfish_ID** | 20 |  |  |  |
| **Fixed Effects** | | | | |
|  | Estimate | Std. Error | z value | p value (>\|z\|) |
| Intercept | 5.0873 | 0.4225 | 12.041 | < 2e-16 |
| Blowing | 3.0998 | 0.4698 | 6.598 | 4.16E-11 |

**Table S5.** Full table for the model on the **effect of blowing on strike success** in the seabass and seabream experiment.

| **Seabass experiment** | | | | |
| --- | --- | --- | --- | --- |
| **Family** | binomial (logit) |  |  |  |
| **Formula** | Strike_success ~ Blowing + (1 \| Lionfish_ID) | | | |
| **Random Effects** | | | | |
| Groups | Name | Variance | Std. Dev. |  |
| Lionfish_ID | (Intercept) | 1.56E+00 | 1.247 |  |
|  |  |  |  |  |
|  |  |  |  |  |
| **Number of observations** | 167 |  |  |  |
| **Lionfish_ID** | 26 |  |  |  |
| **Fixed Effects** | | | | |
|  | Estimate | Std. Error | z value | p value (>\|z\|) |
| Intercept | 1.3523 | 0.667 | 2.027 | 0.0426 |
| Blowing | -0.201 | 0.8797 | -0.228 | 8.19E-01 |

**Figure S1. Contribution of lionfish and prey to the total change in angle during approaches in the seabream experiment.** The panel on the **left** shows the change in angle of lionfish during an approach, relative to the initial lionfish-prey angle. The panel on the **right** shows the change in angle of prey during an approach, relative to the initial lionfish-prey angle.

Alt text: scatterplot showing the extent to which lionfish (left panel) and prey (right panel) changed their orientation during an approach in the seabream experiment.

**Figure S2. Stills from videos that were used for data collection. Panel A** was taken from one of the videos from the cameras mounted on the side of the arena. These videos were used to determine whether a lionfish was blowing or not. **Panel B** was taken from the central camera mounted above the arena. These videos were used to determine angles and distances during an approach. In both videos, a lionfish is approaching a prey with fully flared pectoral fins. Panel B also shows the two cameras mounted on the side of the arena and some lionfish in the common area. The two screws used to calibrate the software for size estimations are visible in both stills.

Alt text: photos showing screenshots from a video from which variables were scored. The screenshots show a prey and a lionfish, with a clear view of their orientation and offer a real picture of the experimental arena.

**Figure S3. Participation rate of individual lionfish during the seabass experiment.** Each mark on the x axis represents an individual and the blue bars show the number of trials in which they participated every day. Dates are shown in the grey labels.

Alt text: bar chart showing how many trials each individual lionfish participated in on every day that experiments were conducted with seabass. Days are shown in separate panels, lionfish as separate bars on the x axis and the number of trials is shown on the y axis.

**Figure S4. Participation rate of individual lionfish during the seabream experiment.** Each mark on the x axis represents an individual and the blue bars show the number of trials in which they participated every day. Dates are shown in the grey labels.

Alt text: bar chart showing how many trials each individual lionfish participated in on every day that experiments were conducted with seabream. Days are shown in separate panels, lionfish as separate bars on the x axis and the number of trials is shown on the y axis.
